# Effect of synthesis conditions on the porous texture of activated carbons obtained from Tara Rubber by FeCl_3_ activation

**DOI:** 10.1038/s41598-024-52112-5

**Published:** 2024-01-27

**Authors:** Mirosław Kwiatkowski, Carolina Belver, Jorge Bedia

**Affiliations:** 1 Department of Fuel Technology, Faculty of Energy and Fuels, AGH University of Krakow, al. Adama Mickiewicza, 30, 30-059 Krakow, Poland; 2 Departamento de Ingeniería Química, Facultad de Ciencias, Universidad Autónoma de Madrid, Campus Cantoblanco, 28049 Madrid, Spain

**Keywords:** Chemical engineering, Physical chemistry, Characterization and analytical techniques

## Abstract

This paper presents the results of an unique analysis of the influence of the mass ratio of activator FeCl_3_ to precursor and the temperature of the activation process on the formation of the porous structure of activated carbons obtained from Tara Rubber by FeCl_3_ activation. The study used the new numerical clustering based adsorption analysis method and the quenched solid density functional theory, taking into account, among other things, the heterogeneity of the analysed surface which is a new approach rarely used in the analysis of the porous structure of adsorbents. On the basis of the calculation results, it was concluded that the activated carbon with the most developed porous texture was obtained at a mass ratio (FeCl_3_:Tara Rubber) of 2, at an activation process temperature of 800 °C. This activated carbon is also characterised by the lowest degree of surface heterogeneity and at the same time, however, the widest range of micropores compared to activated carbons obtained at other mass ratios. The analyses carried out further demonstrated the valuable and complementary information obtained from the structure analysis methods and their high utility in practical applications, especially in the development of new industrial technologies for the production of adsorbents and the selection of optimal conditions for their production.

## Introduction

Owing to their unique properties, such as high specific surface area and pore volume, as well as the substantial advantage of controlling their porous structure through the selection of suitable raw materials and production technologies, microporous carbonaceous adsorbents are in common use in industrial processes and everyday life. Activated carbons are used mainly for adsorption processes^1–5^, although they are also widely used in catalysis, both as mass catalysts^6–9^ or as catalysts supports^10–13^ or in energy applications^14,15^. They are usually obtained by physical activation preceded by carbonisation, or by chemical activation^16^.

Carbonisation is performed by heat treatment at high temperature in an inert atmosphere. During this process, volatiles are removed and char is consequently enriched in elemental carbon^17,18^. The efficiency of the carbonisation process is influenced by the final process temperature, the rate of temperature rise, the inert gas flow rate and the duration of the process^19,20^. The highest mass yield of the carbonisation process is achieved at relatively low temperatures and low heating rates. The char, product of the carbonisation process, has a poorly developed porous structure and a low specific surface area, as the pores are blocked by tarry substances and must therefore undergo an additional physical or chemical activation process^21^. During the above-mentioned processes, the pore-blocking tarry substances are removed and new pores are formed, and consequently, the porous structure, especially the micropores, develops further^22^.

Physical activation can be a one-step process or, more often, a two-step process, i.e. preceded by carbonisation of the raw material, and involves activation of the raw material or char with an oxidising gas at a high temperature, i.e. around 800–1100 °C^23,24^. Carbon dioxide and steam, possibly air (in this case a lower activation temperature is used), are predominantly used as an oxidising gas^23–25^. The extent to which a porous structure develops during physical activation depends mainly on the temperature and duration of the process as well as the reactivity of the gasification agent^26^. The use of carbon dioxide as an activator leads to the formation of predominantly micro- and ultra-microporous structures, while steam activation leads to the development of a wider pore distribution and a higher proportion of mesopores. The main advantages of physical activation include the low cost of producing activated carbons and the ability to retain the shape and texture of the precursor. Physical activation therefore makes it possible to produce low-abrasion activated carbons from hard raw materials such as nut shells and fruit stones, as well as to produce activated nonwovens and fabrics and carbon mats from fibrous precursors^26^.

Chemical activation can, by analogy with physical activation, be a one-step process or a two-step process preceded by carbonisation in the case of obtaining activated carbons from biomass by chemical activation using NaOH or KOH as activator^27–37^, as otherwise these strong bases may dissolve the organic matter of the precursor, making subsequent activation impossible. Compared to physical activation, chemical activation allows better development of surfaces and microporous structures and is less energy-intensive due to the lower process temperature and its shorter time^38,39^. Chemical activation, however, requires an additional washing process of the final product to remove residues of the activator used. Furthermore, from an environmental point of view, hydroxides and alkali metal acids are less favourable as activators because they are highly corrosive and toxic. As a result, other safer activators such as sodium amide NaNH_2_^40^ are increasingly being used alternatively and new methods of producing activated carbons as well as new activators are also being sought and the use of ferric chloride FeCl_3_ as an activator is of particular interest^41^. The use of FeCl_3_ as an activator has a number of advantages over other commonly used activators used in the production of activated carbons, among which are the lower environmental impact as well as the lower cost^41^. This is due to the fact that the use of FeCl_3_ as an activator does not require stringent safety measures and suitable materials for corrosion-resistant plant construction. In addition, a unique feature of activation with FeCl_3_ is the possibility of obtaining activated carbons with very well-developed porosity and a relatively large surface area that are also characterised by magnetic properties^41,42^. In this sense, this study analyses the influence of the mass ratio of activator FeCl_3_ to precursor and the temperature of the activation on the formation of the porous structure of activated carbons obtained from Tara Rubber by FeCl_3_ chemical activation. The precise knowledge of the porosity of activated carbons will help to the improvement of current applications and even to the development of new ones in which a tailored porosity would be needed. In this sense, this research could help to the detailed description of the porosity of activated carbons, providing complementary information about the porosity of these materials.

## Materials and methods

The work of Bedia, and co-workers^42^ presents the unique results of analyses of activated carbons obtained from Tara gum by chemical activation using FeCl_3_ as activator. These activated carbons were obtained at different activation temperatures (400–1000 °C) and at different mass ratios of activator to precursor (FeCl_3_:Tara gum, *r* = 0.5, 1, 2 and 3) obtained by physical mixing. Briefly, initially Tara gum and iron chloride were physically mixed. The mixture was maintained overnight in an oven at 60 °C and then pyrolyzed in a horizontal tube furnace for two hours under continuous N_2_ flow of 150 cm^3^ min^−1^ (n.c.). that was maintained until the furnace was cooled down. It was then washed, firstly with aqueous HCl (0.1 N) at 70 °C during and then with distilled water at room temperature up to neutral pH. The final samples were dried overnight in an oven at 60 °C. The activated carbons were denoted by the letters “GT”, followed by the impregnation ratio and the activation temperature in degrees Celsius (i.e. GT2-800 is the activated carbon prepared with an impregnation ratio of 2 and at an activation temperature of 800 °C). The resulting activated carbons showed a well-developed porous texture, mainly in terms of micropores size, and a superparamagnetic character due to the presence of iron forms in their structure^42^. However, the Brunauer–Emmett–Teller (BET)^43^ and t-plot methods^44^ as well as the Barrett-Joyner-Halenda (BJH) method^45^ for the determination of pore size distributions used in the analysis of porous structure, are criticised for over-simplifying assumptions that deviate significantly from reality without taking into account surface heterogeneities. With the development of the science of physicochemical processes on the surface of solids, significant progress has been made in understanding the mechanisms of adsorption and consequently, a solid theoretical basis for characterising the adsorption process has been developed. This progress is largely related to the application of methods such as Density Functional Theory (DFT), which allows the adsorption and phase behaviour of pore fluids to be described at the molecular level^46^.

The Non Localized Density Functional Theory (NLDFT) method, based on a model of independent gap-shaped pores with ideal graphite walls, has become particularly popular. However, such a model has a significant drawback; starting from a pore width greater than a few molecular diameters, the theoretical adsorption isotherms show many steps associated with layer transitions related to the formation of a monolayer, a second adsorbed layer and so on^47^. Experimentally, however, stepped adsorption isotherms are only obtained at low temperatures for fluids adsorbed on molecularly smooth surfaces. In activated carbons, transitions between layers are hampered by the inherent energetic and geometric heterogeneities of their actual surfaces. The layering steps on the theoretical isotherms cause artificial gaps in the calculated Pore Size Distributions (PSD). Such a discrepancy between the theoretical assumption of a smooth and homogeneous surface and the experimental situation is particularly significant for microporous carbon adsorbents with wide PSDs. The aforementioned problems were solved by the development of a Quenched Solid Density Functional Theory (QSDFT) model that quantifies the geometric heterogeneity of a surface through the roughness parameter^48^. This parameter represents half the width of the range of undulations at the molecular level of the pore wall surface. The aforementioned roughness parameter can be determined by comparing theoretical and experimental adsorption isotherms on the reference surface. The QSDFT method provides a significant improvement over traditional DFT methods as well as the NLDFT method, which assume pore walls as homogeneous graphite-like flat surfaces.

A next novel approach for the analysis of the surface properties of materials is the numerical clustering-based adsorption analysis (LBET) method^49,50^. Compared to the conventional methods, the LBET method considers the heterogeneity of the surface and allows the determination of the size and shape of the adsorbate molecule clusters formed, the adsorption energy distributions (AEDs), and the reliability and credibility of the obtained results^49,50^.

The LBET and QSDFT methods not only provide much more reliable results, but also much more information about the analysed structure. Consequently, the concept of carrying out a new extended cycle of analysis using LBET and QSDFT methods, taking into account, among other things, the heterogeneity of the surface, was developed. The analyses carried out comprehensively considered the effects of activation process temperature and activator/precursor mass ratio (i.e. FeCl_3_:Tara Rubber), on the formation of the porous structure of the resulting activated carbons.

## Discussion of the obtained results

**Figure 1:**
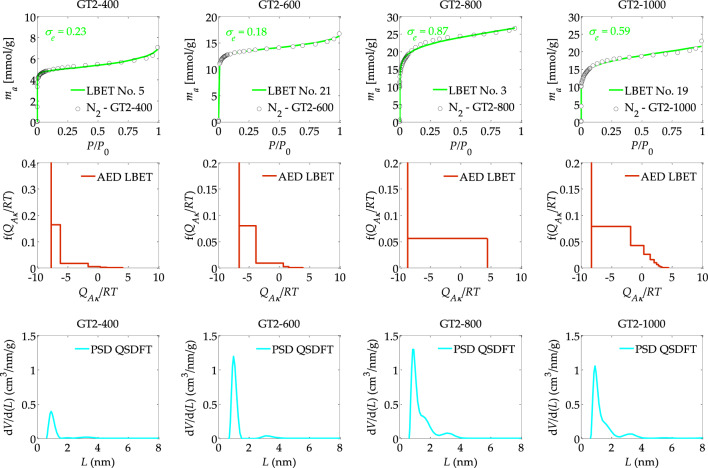
The nitrogen adsorption isotherms and the results of the identification of the adsorption systems via the LBET and QSDFT methods: where AED is the adsorption energy distributions, and PSD is the pore size distributions obtained for the activated carbons prepared via chemical activation using FeCl_3_ as activator at different temperatures of the activation process, i.e. 400, 600, 800 and 1000 °C.

**Figure 2:**
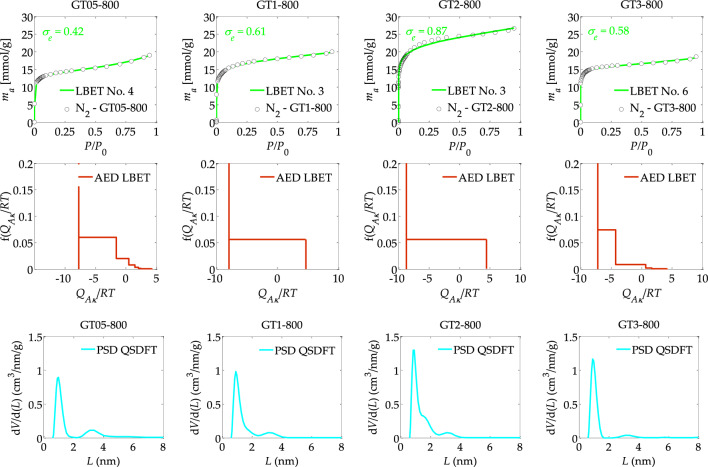
The nitrogen adsorption isotherms and the results of the identification of the adsorption systems via the LBET and QSDFT methods: where AED is the adsorption energy distributions, and PSD is the pore size distributions obtained for the activated carbons prepared via chemical activation using FeCl_3_ as activator at different mass ratios of activator to precursor (FeCl_3_:Tara gum, *r* = 0.5, 1, 2 and 3).

The results of the analyses carried out using the LBET and QSDFT methods are summarised in Tables 1, 2 and shown in Figs. 1, 2. The results of calculations for activated carbon GT2-400, i.e. obtained by chemical activation using FeCl_3_ as activator at a mass ratio of activator to precursor (FeCl_3_:Tara Rubber) equal to 2 and at 400 °C, allow us to conclude that in the micropores of the material studied there are limitations to the growth of nitrogen molecule clusters associated with the competitive expansion of neighbouring clusters, as indicated by the number of the best-fit mathematical model of the adsorption process. The surface of GT2-400 activated carbon is moderately heterogeneous, as indicated by the value of the surface heterogeneity parameter *h* (*h* = 3).

**Table 1:** The results of the analysis of a porous structure of activated carbons prepared via chemical activation using FeCl_3_ as activator at different temperatures of the activation process, i.e. 400, 600, 800 and 1000 °C, based on nitrogen adsorption isotherms, using the LBET and QSDFT methods.

Material	*Model No*	*h*	*V*_*hA*_ [cm^3^/g]	*α*	*β*	*Q*_*A*_/*RT*	*B*_*C*_	*σ*_*e*_	*w*_*id*_	*S*_*QSDFT*_ [m^2^/g]	*V*_*QSDFT*_ [cm^3^/g]	*S*_*BET*_ [m^2^/g]	*V*_*p*_ [cm^3^/g]
GT2-400	5	3	0.157	0.3	3.1	− 7.68	6.8	0.2	0.2	362	0.213	379	0.245
GT2-600	21	3	0.428	0.2	3.1	− 6.54	5.6	0.1	0.5	879	0.517	996	0.582
GT2-800	3	2	1.018	0.7	1.0	− 8.67	6.5	0.8	0.2	1439	0.879	1680	0.990
GT2-1000	19	3	0.601	0.6	1.8	− 8.26	7.7	0.5	0.3	1113	0.702	1298	0.810

*V*_*hA*_ is the volume of the first adsorbed layer, *Q*_*A*_/*RT* is the dimensionless energy parameter for the first adsorbed layer; *B*_*C*_ is the energy parameter for the higher adsorbed layers; *α* is the geometrical parameter of the porous structure determining the height of the adsorbate molecule clusters; *β* is the geometrical parameter of the porous structure determining the width of the adsorbate molecule clusters; *h* is the surface heterogeneity parameter; *S*_*QSDFT*_ is the micropores specific surface area; *V*_*QSDFT*_ is the volume of micropores; *S*_*BET*_ is the BET surface area and *V*_*p*_ is the total pore volume, both obtained for the experimental N_2_ adsorption–desorption isotherms.

**Table 2:** The results of the analysis of a porous structure of activated carbons prepared via chemical activation using FeCl_3_ as activator at different mass ratios of activator to precursor (FeCl_3_:Tara gum, *r* = 0.5, 1, 2 and 3), based on nitrogen adsorption isotherms, using the LBET and QSDFT methods.

Material	*Model No*	*h*	*V*_*hA*_ [cm^3^/g]	*α*	*β*	*Q*_*A*_/*RT*	*B*_*C*_	*σ*_*e*_	*w*_*id*_	*S*_*QSDFT*_ [m^2^/g]	*V*_*QSDFT*_ [cm^3^/g]
GT05-800	4	3	0.463	0.42	3.19	− 7.68	7.40	0.42	0.26	940	0.634
GT1-800	3	2	0.781	0.70	1.00	− 7.93	7.73	0.61	0.13	1075	0.657
GT2-800	3	2	1.018	0.74	1.00	− 8.67	6.55	0.87	0.28	1439	0.879
GT3-800	6	3	0.534	0.26	2.75	− 7.15	7.73	0.58	0.04	1074	0.623

GT2-400 activated carbon has a poorly developed porous structure, as indicated by the value of the volume parameter of the first adsorbed layer *V*_*hA*_ (*V*_*hA*_ = 0.157 cm^3^/g) and the values of the *S*_*QSDFT*_, *V*_*QSDFT*_ parameters, *i.e.* specific surface area and pore volume, respectively, determined using the QSDFT method (*S*_*QSDFT*_ = 362 m^2^/g, *V*_*QSDFT*_ = 0.213 cm^3^/g). In the pores of this material, low and significantly branching clusters of nitrogen molecules form, as indicated by the values of the geometrical parameters of the LBET model, i.e. the heights of the adsorbate clusters *α* and their width *β* (*α* = 0.34, and *β* = 3.10). In turn, the values of the energy parameters *Q*_*A*_*/RT* and *B*_*C*_ indicate preferential conditions for the multilayer adsorption process (*Q*_*A*_*/RT* =  − 7.68 and *B*_*C*_ = 6.83).

It is noteworthy that, despite a very good fit of the theoretical isotherm to the empirical isotherm, as indicated by the dispersion value of the fit error *σ*_*e*_ = 0.23, the value of the traceability parameter *w*_*id*_ (*w*_*id*_ = 0.29), may indicate the presence of some deviations of the actual structure from the assumptions made in the structure model implemented in the LBET method. Another material analysed was the GT2-600 activated carbon sample obtained at a mass ratio of *r* = 2, at an activation temperature of 600 °C in contrast to the previously analysed GT2-400 activated carbon, this material shows geometric limitations to the growth of clusters of adsorbate molecules as indicated by the number on a better-fitting cluster mathematical model of the adsorption process.

Activated carbon GT2-600 is characterised by moderate surface heterogeneity, similar to activated carbon GT2-400. However, the higher value of the *V*_*ha*_ parameter, i.e. the volume of the first adsorbed layer (*V*_*ha*_ = 0.428 cm^3^/g), as well as the *S*_*QSDFT*_ and *V*_*QSDFT*_ parameters (*S*_*QSDFT*_ = 879 m^2^/g and *V*_*QSDFT*_ = 0.517 cm^3^/g), indicates a greater development of the microporous structure. The value of the geometrical parameter for the height of clusters of adsorbate molecules *α* is, however, lower (*α* = 0.26) compared to the previously analysed material, indicating the formation of lower clusters of nitrogen molecules in the micropores of activated carbon GT2-600 compared to activated carbon obtained at a lower temperature, and thus the greater presence of small micropores in the pore structure of this material. In the case of sample GT2-600, the very good identifiability of the adsorption system, and thus the high reliability of the determined porous structure parameters, is noteworthy.

The next activated carbon sample analysed, designated GT2-800 and therefore obtained at a mass ratio of *r* = 2 and at an activation temperature of 800 °C, is characterised by limitations on the expansion of nitrogen molecule clusters due to the competitive expansion of neighbouring clusters. The sample is also characterised by the lowest degree of surface heterogeneity among the analysed activated carbons obtained at an impregnation mass ratio of 2. Activated carbon GT2-800 also had the most developed porous structure, as indicated by the very high value of the volume of the first adsorbed layer *V*_*hA*_ (*V*_*hA*_ = 1.018 cm^3^/g), the very high value of the *S*_*QSDFT*_ and *V*_*QSDFT*_ parameters (*S*_*QSDFT*_ = 1439 m^2^/g, and *V*_*QSDFT*_ = 0.879 cm^3^/g) and the high height of the clusters of adsorbate molecules, as indicated by the value of the geometrical parameter *α* (*α* = 0.74). In turn, the value of the geometrical parameter *β* indicates that non-branching clusters of adsorbate molecules are formed in the pores of the analysed sample. Of note is the average identifiability of the N_2_—GT2-800 adsorption system, indicating the presence of some deviations from the theoretical model of the porous structure from the actual pore structure of the analysed material.

Analysis of a further sample of GT2-1000 activated carbon obtained at an activator/precursor mass ratio of 2 and an activation temperature of 1000 °C, showed limitations in the growth of clusters of adsorbate molecules associated with geometrical pore limitations and destruction of the porous structure associated with a too high activation temperature, as indicated by significantly lower values of the *V*_*hA*_ parameters, *S*_*QSDFT*_ and *V*_*QSDFT*_ parameters and *α* in comparison with the values of these porous structure parameters determined for GT2-800 activated carbon obtained at a lower temperature, i.e. 800 °C. The increase of the activation temperature from 800 to 1000 °C produces a significantly deeper devolatilization of the organic precursor. This results in an increase of the ash content of the sample^42^ and consequently to a decrease in the porosity of the sample, since ashes are essentially non-porous.

The shapes of the graphs of AED adsorption energy distributions on the surface of the analysed activated carbons determined by the LBET method, shown in Fig. 1, indicate the occurrence in the analysed samples of a significant proportion of sites with equal energy, i.e. micropores in which single nitrogen molecules adsorb, and the occurrence of a significant proportion of sites with a wide range of adsorption energies.

It is also noteworthy that activated carbon GT2-800, in contrast to the other analysed samples obtained at a mass ratio of *r* = 2, is characterized by a uniform distribution of sites with a wider energy range and thus the lowest surface energy heterogeneities. Pore size distributions were also determined for the carbons analysed using the QSDFT method, the shape of which indicates the predominant share of micropores in the total porosity of the activated carbons analysed, and some small share of small mesopores. It is noteworthy the smallest volume of micropores obtained for sample GT2-400 (see Fig. 1).

The study also analysed the effect of the mass ratio of activator to precursor on the formation of the porous structure of activated carbons obtained from Tara Rubber using FeCl_3_ as activator at mass ratios from *r* = 0.5–3. The results of the analyses using the LBET and QSDFT methods are shown in Table 2 and Fig. 2.

Analysis of the results obtained for GT05-800 activated carbon showed that the limitations of nitrogen molecule cluster expansion are due to the competitive expansion of neighbouring clusters. The surface area of GT05-800 activated carbon is moderately heterogeneous, as indicated by the value of the parameter *h* = 3, and the values of the parameters *V*_*hA*_, *α*, *S*_*QSDFT*_, *V*_*QSDFT*_ (*V*_*hA*_ = 0.463 cm^3^/g, *α* = 0.42, *S*_*QSDFT*_ = 940 m^2^/g, *V*_*QSDFT*_ = 0.634 cm^3^/g) indicate good development of the porous structure.

The value of the geometrical parameter of the cluster width *β*, indicates the presence of significantly branched clusters of nitrogen molecules (*β* = 3.19) in the pores of the analysed material. The values of the energy parameters *Q*_*A*_*/RT* and *B*_*C*_ (*Q*_*A*_*/RT* = − 7.68 and *B*_*C*_ = 7.40) indicate preferential conditions for the multilayer adsorption process to take place. Only the average fit of the theoretical isotherm to the empirical data is noteworthy, and thus also the average traceability of the absorption system indicating the occurrence of some deviations of the porous structure from the assumed model pore structure in the LBET method.

The next sample analysed was an activated carbon sample designated GT1-800, i.e. a material obtained at an activator/precursor (FeCl_3_:Tara Rubber) mass ratio of 2, at an activation temperature of 800 °C. This sample is characterised by a lower degree of surface heterogeneity as indicated by the value of the surface heterogeneity parameter *h* (*h* = 2) as well as a more developed porous structure as indicated by the values of the parameters *V*_*hA*_, *α*, *S*_*QSDFT*_ and *V*_*QSDFT*_. High non-branching clusters of adsorbate molecules form in the pores of the activated carbon, as indicated by the values of the geometric parameters *α* and *β* (*α* = 0.70, and *β* = 1.00).

The results of the analysis of a further GT2-800 activated carbon sample obtained at a mass ratio of 2, at an activation temperature of 800 °C, which has already been described in detail above. In summary, this sample shows the most developed microporous structure, as indicated by the highest values of the *V*_*hA*_, *α*, *S*_*QSDFT*_ and *V*_*QSDFT*_ parameters, of all the activated carbons analysed throughout the study cycle. Further increasing the mass ratio of activator to precursor, i.e. to *r* = 3 in the case of activated carbon GT3-800 resulted in the destruction of the porous structure, as indicated by significantly lower values of the *V*_*hA*_ parameters, *α*, *S*_*QSDFT*_ and *V*_*QSDFT*_.

The shapes of the energy distribution diagrams of AED adsorption on the surface of the analysed activated carbons determined by the LBET method, shown in Fig. 2, indicate the predominance of sites with equal energy in all activated carbon samples, which allows the presence of micropores in which single nitrogen molecules adsorb to be inferred. Activated carbon GT3-800 has, as can be seen, the narrowest adsorption energy distribution on the surface. On the other hand, the analysis of the pore size distributions determined for the activated carbons analysed and presented in Fig. 2, indicate that the average size of the micropores contributed to the total porosity of the activated carbons analysed.

## Conclusions

The analytical results presented here are a significant and unique extension of previous studies and have provided a lot of new reliable information on activated carbons obtained at different activation temperatures and at equal mass ratios, thus providing the opportunity to determine the optimum process conditions for producing an activated carbon dedicated to a specific adsorption process. On the basis of the study, it was shown that, among the various synthesis conditions, the activation temperature and, to a lesser extent, the mass ratio of activator to precursor are the most significant parameters affecting the final properties of the activated carbons obtained. On the basis of the calculation and analysis results obtained, it was shown that activated carbon with the highest porous structure development was obtained at a mass ratio (FeCl_3_:Tara Rubber) of 2, at an activation process temperature of 800 °C. This activated carbon is also characterised by the lowest degree of surface heterogeneity and at the same time, however, the widest range of micropores compared to activated carbons obtained at other activator/precursor mass ratios. The analysis also demonstrated the mutual complementarity of the LBET and QSDFT methods, which, through their simultaneous application, enable a comprehensive and reliable analysis of the porous structure taking into account, among other things, surface heterogeneities. This method, together with appropriate economic analysis, helps the precise selection of the method and conditions of the process of obtaining activated carbons at specific costs of the manufacturing process, and thus makes it possible to obtain materials that can successfully compete with other technologies used in industrial practice and everyday life.

## Data Availability

All data generated or analysed during this study are included in this published article.
